# Our Correspondence

**Published:** 1871-04

**Authors:** 


### Our Correspondence.

OIL CITY, Pa., Jan. 16, 1871.

THAD S. UP DE GRAFF, M. D.—

*Dear Sir*:—I have received your BISTOURY for some time and like it very much. I have for the last two months been using *Iodoform* in the treatment of constitutional syphilis; in the mean time it has cured two cases of chronic inflammation of the prostate gland; one case was of six and the other of sixteen months standing. Have you known iodoform to be used for this purpose?

THAD. W. EGBERT, M. D.

We have not known of iodoform being used for inflammation of the prostate gland. Dr. Egbert's hint may be useful to the profession.

MIDWAY, Ala., Sept. 12, 1870.

THAD S. UP DE GRAFF, M. D.—

*Dear Sir:*—I want you to give M. Wagner & Co., of Marshall, Mich., a slight notice in your excellent little journal. In April last, for the sum of $10, as a guarantee of honest dealing on my part, the above firm, (so called,) sent me 70 family rights to manufacture the celebrated Dr. Culver's "Sunlight Oil," with a certificate of Agency for Bullock County, Ala.; giving to me the exclusive right to the county, saying in that certificate that no other agencies would be granted in the same county, and no other person allowed to trespass on my territory. In less than one month, another agent for the same county came to my house to sell me a family right for the same thing.

I had made some of this "Sunlight Oil," and upon trial, found it so inflamable and so very explosive, that I considered it dangerous, and made no effort to sell any of it. Since the agent above spoken of came here, I have found two others for the same county, making four agents within my knowledge for the same county, each with a certificate, securing the county to him alone. This is a new dodge for picking up the extra X's a man has about him, and I want the scoundrels published, that others may know of their manner of doing business, and the danger of the oil which they are having manufactured throughout the country.

Respectfully Yours, &c

B. F. COLEMAN, M. D.

We wonder that there is no way of reaching such scoundrels. Swindling their Agents is a small affair in comparison to the danger to families from the murderous compound which they seek to distribute throughout the country. We advise people to beware of any patent compound sold them for "non-explosive oil."

---

Were we given to slang phrases we would find a great deal of satisfaction in inquiring—"How is this for high?" Read it and imagine if you can, what sort of a "Doctor" penned it. Is it not astonishing that people can be found who can trust their lives in the hands of such illiterate blunderers? Read the effusion—it runs as follows:—

PENN'S CREEK, SNYDER Co., Pa.

MR. UP—

*Sir*:—As I have heard of your BISTORY I thot That I would give you a account of Obstitrics case that I met with in my Practice this was a Mrs. H. that I was called to see in her first confinement on Oct. 27, 1869 and on Nov. 30, 1870 I was called to see her again beind 13 months and one Day about and she gave Birth to a Mail child hiving To Teeth incisors and the child is dong well.

This is the first case that of that Kind that has ever come under my Practice.

Yours Truly,
J. F. K.

---

FREEPORT, Ills., Feb. 20, 1871.

EDITOR OF BISTOURY.—

I often hear traveling agents for Eastern maufacturers of Elixirs, say, "we do not trust our goods to physicians, but get them to recommend the druggist to purchase," &c.

This slur upon the profession, reminds me of my experience for the past two years, which I will give in brief. I have had dealings with sixteen thousand one hundred and seventy-one physicians, and every one of them has paid me promptly at time of maturity, with but three exceptions. And with this experience I am fully convinced that the true physician is of all men, most worthy of credit.

Truly Yours,
L. A. BABCOCK, M. D.

---

HISEVILLE, Ky., Dec. 15, 1870.

DR. T. S. UP DE GRAFF, ELMIRA, N. Y.—

In the last number of your paper, I noticed the mention of two physicians relative to the use and success of Babcock's Uterine Supporter in the treatment of diseases of the uterus. In corroboration of their testimony, I beg leave to report the very happy success in the use of one of the instruments myself. My patient, Mrs. —, had been suffering for several years with procidentia and ulceration of the uterus, and from the congested and hypertrophied state of the organ, she suffered much and long continued pain, frequently followed by excessive and indeed, almost uncontrollable uterine hemorrhage. I resorted to use of the means which I could command, with the aid of the consultation of the oldest and best physicians in my county, time and expense being no object in her case, using the very best and most highly recommended pessaries, but all seemed to give her but momentary relief. In this condition, so unsatisfactory to us all, I was induced, from a close examination of Babcock's Uterine Supporter, to try it in her case. And since then I have heartily blessed the hour in which the thought was conceived. I immediately procured and applied the instrument, and to my great satisfaction she has been improving ever since. The organ is now in its proper position, the ulceration has entirely subsided, she voids urine without the use of the catheter, and those severe and alarming hemorrhages have not occurred since she commenced the use of the instrument. My patient can now walk about the room and go visiting in her buggy without any inconvenience at all. She says, "I can't do without it; I would not part with it, if I could not get another, for a thousand dollars." I believe it to be the best instrument in use for the purposes intended.

Yours Truly,
S. L. NEWBERRY, M. D.

---

DEAR DOCTOR,—

I like your journal much. I enclose a prescription for improved Dover's Powder, which if you like, you may place among your valuable formulary.

℞.

Pulv. Camphoræ, ʒ ij;

Pulv Opii, ʒi;

Pulv. Ipicac., ʒ j;

Graulated Sacch. Alb, ℥ss. M.

Mix. G. B. C.

---

ROCHESTER, N. Y. Feb. 20, 1871.

THAD S. UP DE GRAFF, M. D.—

*Dear Sir:*—I have taken your BISTOURY for two years, and regard it as my most valuable medical journal. Its "Medical Items and News"

is alone worth ten times what you ask for your Magazine. I have discarded several medical journals and rely upon the BISTOURY to *condense* for me, in two or three pages, what it costs me much time and money to procure. You are doing a good work in exposing the various advertised medical swindles of the day, and I wish you success. I send you a club from this place, hoping in this manner to exhibit my appreciation of your work, and in a small way to assist in increasing your usefulness.

Fraternally Yours,

E. L. W., M. D.

---

MIDDLETOWN, PA., Jan 30, 1871.

DR. UP DE GRAFF.—

*Dear Sir:*—I have been a reader of your Magazine for the past year, and most sincerely wish I had known of it sooner—it would have saved me considerable money and my family from much grief.

I have just taken our only son to an insane asylum, who lost his mind partly from vice, but more particularly from reading the abominable quack literature which he found advertised in the city and country newspapers. Everything he saw advertised, which he imagined had a bearing upon his case, he bought, until he was frightened into believing that it was necessary to patronize every advertising swindler who sent him circulars and medicines. He has been a victim of the "Rev. Chas E. King"—"Sayer & Co.,"—"Rev. Herman Stafford"—"Jos. T. Inman"—"Howard Association," &c., &c., and has taken medicine from all of them until he has lost his health and mind. I have read your warning repeatedly in the BISTOURY, and desire to add my testimony to the truth that you have written concerning the above mentioned advertising scoundrels. I also desire to warn parents against allowing their sons to read such abominable trash as will be found advertised in almost every newspaper—under the title of the "Bridal Chamber"—"Manhood, how lost and how restored"—"Hints to young men," &c., &c. They were the ruination of my boy and I doubt not have ruined very many more.

Truly Yours,

J. H. M.

---

OWEGO, N. Y. Feb. 20, 1871.

EDITOR BISTOURY.—

I read your journal and can testify to the truth of what you have written about advertising doctors. I was unfortunate in contracting a bad disease. Was ashamed to go to our family physician as I should have done, but wrote to a New York man who advertised to cure such diseases. He sent me medicine for which I paid him $50. I got no better--he sent me more—still I got worse until I am now a cripple in my father's house, and my doctor tells me I am likely to remain so. I write this as a warning to young men to let the advertising doctors alone.

Yours, &c.,

A CRIPPLE.

---

ADAMS, N. Y., Feb. 24, 1871.

EDITOR OF BISTOURY:—

Being on the wing most of the time, I have frequent opportunities for seeing "the doings" of traveling medical humbugs, a class of rascals you are so capable of flaying alive. By the way, there is one of that class you seem to have overlooked; at least I do not remember of ever seeing him mentioned in your journal. He is a very pretentious scamp, and is "pocketing the stamps" in a manner exceedingly gratifying to himself. He was a resident of your little city several years ago, and claims to have conducted the Commercial College there. While engaged in that honest vocation he discovered that he was endowed with great healing powers. By simply "laying on his hands," or by rubbing; diseases hitherto intractable to treatment, disappeared as by the touch of the magician's wand. The blind were made to see, the deaf to hear, the lame to walk; and many other wonderful things were done through his manipulations. In short, he found out that he was what you call a "buster," and so he concluded to travel, that he might benefit suffering humanity, and replenish his nearly exhausted finances. The latter object he has probably accomplished, judging by the number of five dollar miracles I have heard of his working. His fees are pretty high, yet I see that he is not devoid of generosity, for in his circular he announces that "the poor *widows* will be kindly considered."

Notwithstanding the many certificates of his wonderful cures which he has published—not even that one appertaining to the complete restoration of a girl twenty-one years old who was born blind—I am not a believer in his great healing powers. Indeed I have known many afflicted ones to take his "treatment," and have yet to see one case that was cured by him. Those who do not wish to admit that they have been humbugged, think he has helped them, but others confess that they have been duped and denounce the rogue.

Now, dear BISTOTRY, will you not give this vagabond a mighty "lift" in your next number, for I do not know a quack who more richly dedeserves it. Do you know anything about the big cures he publishes as having been done at Elmira, or do you know where he received the fine medical education of which he boasts?

Yours,

D. S., JR.

The idea of any one employing such an abominable "fraud" as the one above mentioned, is so ridiculous that we had no fear of people being swindled by him. All we have to say with reference to him is, that people who are silly enough to pay him money for his absurd "hocus-pockus-laying-on-of-hand-cure," richly deserve being humbugged.

### COUNT MOLTKE, AGED 70.

The most potential man in the world just now is General Moltke, and the days of his years are threescore years and ten. We will leave military critics to do justice to the military genius of Moltke, and to say where he is to be placed in comparison with Grant, and Wellington, and Napoleon, and Marlborough and the older heroes of the world. What we design now is much more simple, but equally interesting. The "still strong man," about whom one hears so little, who can be "interviewed" only by Bismarck and by the royal family of Prussia, and without whom all Bismarck's grand designs might have been unavailing, the man who is renewing the art of war, and concentrating with such terrible efficiency the whole force and manhood and discipline of Germany, is seventy years old. The King of Prussia, himself seventy-three, has made him a count in honor of his seventieth birthday; but to us it is far more interesting to know that he has reached that age, than to hear that he has become Count Moltke. Grant is not yet fifty years old. Marlborough was all done with war by the time he was about sixty. Napoleon died at the age of fifty-two. Wellington's military career was over before the age at which Moltke began to distinguish himself. Indeed, before the war with Austria, Moltke has kept his power and his genius very much to himself.

Here, then, is a point for physiologists, that a man of seventy may alter the complexion of the world, and the relation of nations, and the history of civilization; that he may at this age have physical power for going through arduous bodily exertion, and mental power for solving the most tremendous military problems. Meantime let the example of Moltke cheer old men and, make many young men more modest.—*Lancet.*

